# Validating machine learning approaches for prediction of donor related complication in microsurgical breast reconstruction: a retrospective cohort study

**DOI:** 10.1038/s41598-021-85155-z

**Published:** 2021-03-10

**Authors:** Yujin Myung, Sungmi Jeon, Chanyeong Heo, Eun-Kyu Kim, Eunyoung Kang, Hee-Chul Shin, Eun-Joo Yang, Jae Hoon Jeong

**Affiliations:** 1 Department of Plastic and Reconstructive Surgery, Seoul National University Bundang Hospital, 82, Gumi-ro 173 Beon-gil, Bundang-gu, Seongnam-si, Gyeonggi-do 13620 Republic of Korea; 2 Department of Surgery, Seoul National University Bundang Hospital, Seongnam, Republic of Korea; 3 Department of Rehabilitation Medicine, Seoul National University Bundang Hospital, Seongnam, Republic of Korea

**Keywords:** Medical research, Oncology, Risk factors

## Abstract

Autologous reconstruction using abdominal flaps remains the most popular method for breast reconstruction worldwide. We aimed to evaluate a prediction model using machine-learning methods and to determine which factors increase abdominal flap donor site complications with logistic regression. We evaluated the predictive ability of different machine learning packages, reviewing a cohort of breast reconstruction patients who underwent abdominal flaps. We analyzed 13 treatment variables for effects on the abdominal donor site complication rates. To overcome data imbalances, random over sampling example (ROSE) method was used. Data were divided into training and testing sets. Prediction accuracy, sensitivity, specificity, and predictive power (AUC) were measured by applying neuralnet, nnet, and RSNNS machine learning packages. A total of 568 patients were analyzed. The supervised learning package that performed the most effective prediction was neuralnet. Factors that significantly affected donor-related complication was size of the fascial defect, history of diabetes, muscle sparing type, and presence or absence of adjuvant chemotherapy. The risk cutoff value for fascial defect was 37.5 cm^2^. High-risk group complication rates analyzed by statistical method were significant compared to the low-risk group (26% vs 1.7%). These results may help surgeons to achieve better surgical outcomes and reduce postoperative burden.

## Introduction

Ever since Hartrampf first introduced breast reconstruction using transverse abdominal flaps to the world in 1982^1^, abdominal flap breast reconstruction including transverse rectus abdominis muscle (TRAM) flap and deep inferior epigastric artery perforator (DIEP) flap has become a mainstay technique among autologous breast reconstruction surgeons, and has become one of the most popular methods worldwide^2,3^. However, as with all autologous reconstruction methods, morbidity after abdominal flap surgery cannot be avoided. One of the most troublesome complications related to the donor site would be postoperative weakness of the abdominal wall and rectus muscles caused by damage to the deep fascia and the rectus muscles themselves during flap harvesting^4–7^. These complications greatly affect the patient's quality of life after surgery^8^.

Techniques for elevation of the abdominal flap have evolved to reduce morbidity of the donor site as much as possible. Examples include adjustments to the classic TRAM flap, muscle sparing TRAM flap, and DIEP flap^5^. Each technique still maintains unique risks however, such as in the case of DIEP elevated with a single perforator, where the risk of fat necrosis is high when a large flap volume is harvested^9,10^. In attempts to mediate this risk, surgeons try to harvest more perforator vessels, but this in turn leads to a larger fascial defect. There have been numerous studies regarding the risks of abdominal complication and defect closure in abdominal flap surgeries that state conflicting conclusions^4–7,11,12^.

In addition, since complication rates of the donor part after operation are not very high, and typical symptoms, such as abdominal bulging or hernia diagnosed mainly through subjective complaints of patients, a large patient cohort study with follow-up through unified standards and methods is difficult to establish. Also, even if such a study were possible, the data may be severely imbalanced due to the low prevalence rates of individual complication types. It would thus, be difficult to evaluate multiple factors regarding flap elevation and the wound closure process at the donor site.

Machine learning can augment existing statistical processing methods to provide a more accurate and efficient prediction through data obtained in real life, including various clinical situations^13^. Machine learning is a subfield of artificial intelligence in computer science that has evolved from the study of pattern recognition and computer learning theory. It is a technology that studies and builds a system that learns based on empirical data, performs predictions, and consistently improves on its own performance by modifying its algorithms. Rather than executing strictly defined static program instructions, machine learning algorithms build internal models to derive predictions or decisions based on input data. In the process of establishing a machine learning prediction model in creating the algorithm, it is important to sample a wide variance of data within a range that does not harm overall data integrity^14–17^. Using machine learning, we aimed to evaluate factors responsible for donor-related complication, and to evaluate the odds ratio between high versus low risk cohorts to provide more accurate and detailed information for patients in the process of planning, performing, and following up cases of TRAM flap surgeries.

## Methods

### Patients

A retrospective cohort study was conducted for patients who underwent muscle-sparing type TRAM and DIEP (muscle sparing type 3) flaps for immediate or delayed unilateral breast reconstruction at Seoul National University Bundang Hospital from 2006 through 2019. All study participants provided informed consent and the study design was approved by the institutional review board.

## Data collection

A comprehensive review was conducted on electronic medical records, clinical data warehouse systems of our institution, and radiographic imaging studies. A total of 13 factors related to muscle sparing TRAM flap elevation and donor closure were investigated including: age, body mass index, smoking status, prediagnosed diabetes mellitus or hypertension, history of open surgery, history of abdominal laparoscopy or endoscopic surgery, preoperative chemotherapy, elevated flap weight, muscle sparing type, size of fascia defect after flap elevation, and closure pattern of fascia during donor closure (primary closure, nonabsorbable mesh assisted closure, acellular dermal matrix assisted closure). If the patient complained of abdominal bulging or hernia during the outpatient follow-up process, a complication was present, regardless of the severity of symptoms or corrective surgery.

### Statistical methods

For this study, the R program version 3.6.3 (The R Foundation for Statistical Computing, Vienna, Austria) was used. Installed and used packages were: mlbench, moonBook, random over sampling example (ROSE), rpart, neuralnet, Metrics, pROC, ROCR, nnet, devtools, and RSNNS. Logistic regression was performed to investigate the effect of each factor on donor-related complications. Risk factors affecting abdominal complication were investigated among 15 variables by logistic regression, and such selection was determined according to statistical significance. Missing values were excluded. Statistical significance was set at p < 0.05. Goodness of fit was evaluated through the Hosmer–Lemeshow test. AUC (area under curve) was measured through the ROC (receiver operating characteristic) curve.

### ROSE: sMOTE oversampling

As the imbalance of clinical data in present study was severe due to an extremely low complication rate, ROSE oversampling was performed using a method previously published^17^. In cases of applying machine learning to real world data, there are several ways to solve for class imbalances, defined as an overly large discrepancy weighted towards one class. There are methods such as class weight, which gives heavier cost to minority classes; down-sampling, which subtracts randomly from the majority class; and up-sampling, which randomly replicates minority classes. The ROSE package used by the authors is a synthetic minority sampling technique that interpolates from minority classes while down sampling the majority class^18^.

### Neuralnet

The neuralnet package has very flexible functions for training feed-forward neural networks^19^. To approximate the functional relationship, this package can theoretically process covariates and response variables as well as any number of hidden layers and hidden neurons. Although computational cost can increase exponentially with complexity, there is also an option to stop the iteration at any point. The maximum number of iteration steps can be defined by the user and is reached before the algorithm converges. In addition, the package provides functions to visualize results, and generally includes several functions to facilitate the use of neural networks. Total of six neurons were utilized repeatedly, with resilient backpropagation with backtracking. Error function was sum of squared errors and logistic function with threshold of 0.08 was applied. Stepmax was set at 1e6, as the result yielded better results than 1e5.

### NNET

The nnet package is also a widely used neural network package because both categorical data and continuous variables can be classified simply without any additional work^20^. Like neuralnet, it works using a single thread and is a relatively simple package by recent standards without any significant GPU requirements. Furthermore, although the basic functional process itself is simple, adding more hidden layers does not significantly lead to increased additional computational cost or GPU usage frequency. One hidden layer was applied, with two different set of bias progression. The number of total hidden nodes (size) was set at 6, repeating maxit was set at 500, decay weight parameter was applied in order to prevent overfitting of the predictive results. Different subsets were recategorized in order to yield final sensitivity and specificity, by creating total of four different subsets.

### RSNNS: neural networks using the stuttgart neural network simulator (SNNS)

SNNS is a library, containing many standard implementations of neural networks. It provides several different neural network and visualization functions, including multi-layer perceptrons (MLP) capabilities^21^. The RSNNS package wraps SNNS functionality to make it available from within the R program. Using the RSNNS low-level interface, the algorithmic functionality and flexibility of SNNS can be accessed. Furthermore, the package contains a convenient high-level interface, so that the most common neural network topologies and learning algorithms integrate seamlessly into the R program. Total of 500 train data and 68 test data was utilized, additionally categorized into input and output datasets in order to perform multi-layer perceptron function. Total learning number (maxit) was set to 1000 in order to achieve more predictive power.

### Risk cutoff point

By separating factors identified as a direct cause of donor-related complication risk, the optimal reference point representing the risk of complication risk was searched through the ROC curve through the Youden index method, and data was classified above and below the cutoff point^22^. Following the classification, the risk assessment was conducted separately by dividing the high-risk group and the low-risk group.

## Results

A total of 568 patients who underwent muscle sparing TRAM operation for breast reconstruction were enrolled in this study. Patient demographics and variables were summarized in Table 1. Mean follow up period was 68 months. Among the patients, a total of 37 (6.5%) patients complained of abdominal bulging and confirmed with physical examination by the surgeon who operated on, of which 12 (2.1%) were radiographically diagnosed abdominal hernia, and 7 (1.2%) required hernia repair surgery.

**Table 1:** Demographics and logistic regression results of major variables. (*p < 0.05).

Variable	Total n = 568	Donor complication ( +)	Donor complication (−)	p-value	Odds Ratio	95% CI*
n	568	37	531			
**Age**	48.7 (sd 8.7)	48.1	48.8	0.6		
**Body mass index (kg/m**^**2**^**)**	25 (sd 4.4)	25	25	0.84		
**Smoking status**						
Smoker	12 (2%)	1 (3)	11 (2)	0.6		
Non-smoker	556 (98%)	36 (97)	520 (98)			
**Diabetes**						
Yes	21 (4%)	9 (24)	12 (2)	< 0.01*	**1.71**	**1.49–2.16**
No	547 (96%)	28 (76)	519 (98)			
**Hypertension**						
Yes	75 (13%)	6 (16)	69 (13)	0.68		
No	493 (87%)	31 (84)	462 (87)			
**Flap weight**	670 g (sd 92.3)	682	669	0.54		
**Fascia defect size**	33.5 (sd 11.7)	39.8	33	< 0.01*	**1.62**	**1.11–2.04**
**Closure method**						
Simple	197 (35%)	12 (32)	185 (35)	0.7		
Mesh	191 (34%)	9 (24)	182 (34)			
ADM	180 (31%)	16 (43)	164 (31)			
**Cesarean section history**						
Yes	203 (36%)	19 (51)	184 (35)	0.42		
No	365 (64%)	18 (49)	347 (65)			
**Laparoscopy history**						
Yes	79 (14%)	5 (14)	74 (14)	0.51		
No	489 (86%)	32 (86)	457 (86)			
**Neoadjuvant chemotherapy**						
Yes	71(13%)	12 (32)	59 (11)	0.19		
No	497(87%)	25 (68)	472 (89)			
**Adjuvant chemotherapy**						
Yes	280 (49%)	26 (70)	254 (48)	< 0.01*	**2.1**	**1.87–2.54**
No	288 (51%)	11 (30)	277 (52)			
**Muscle sparing type**						
0	111 (20%)	9 (24)	102 (19)	0.03*	**1.35**	**1.02–1.51**
1	191 (34%)	11 (30)	180 (34)			
2	147 (26%)	10 (27)	137 (26)			
3	118 (21%)	7 (19)	111 (21)		**0.20**	**0.06–0.76**

*CI* confidence interval.

Among the different treatment variables analyzed through multivariable logistic regression, the factors that significantly affected occurrences of abdominal complication were: large fascial defect size (OR 1.62), muscle sparing type 0 (OR 1.35), diabetes mellitus (OR 1.71), and adjuvant chemotherapy (OR 2.1).

**Figure 1:**
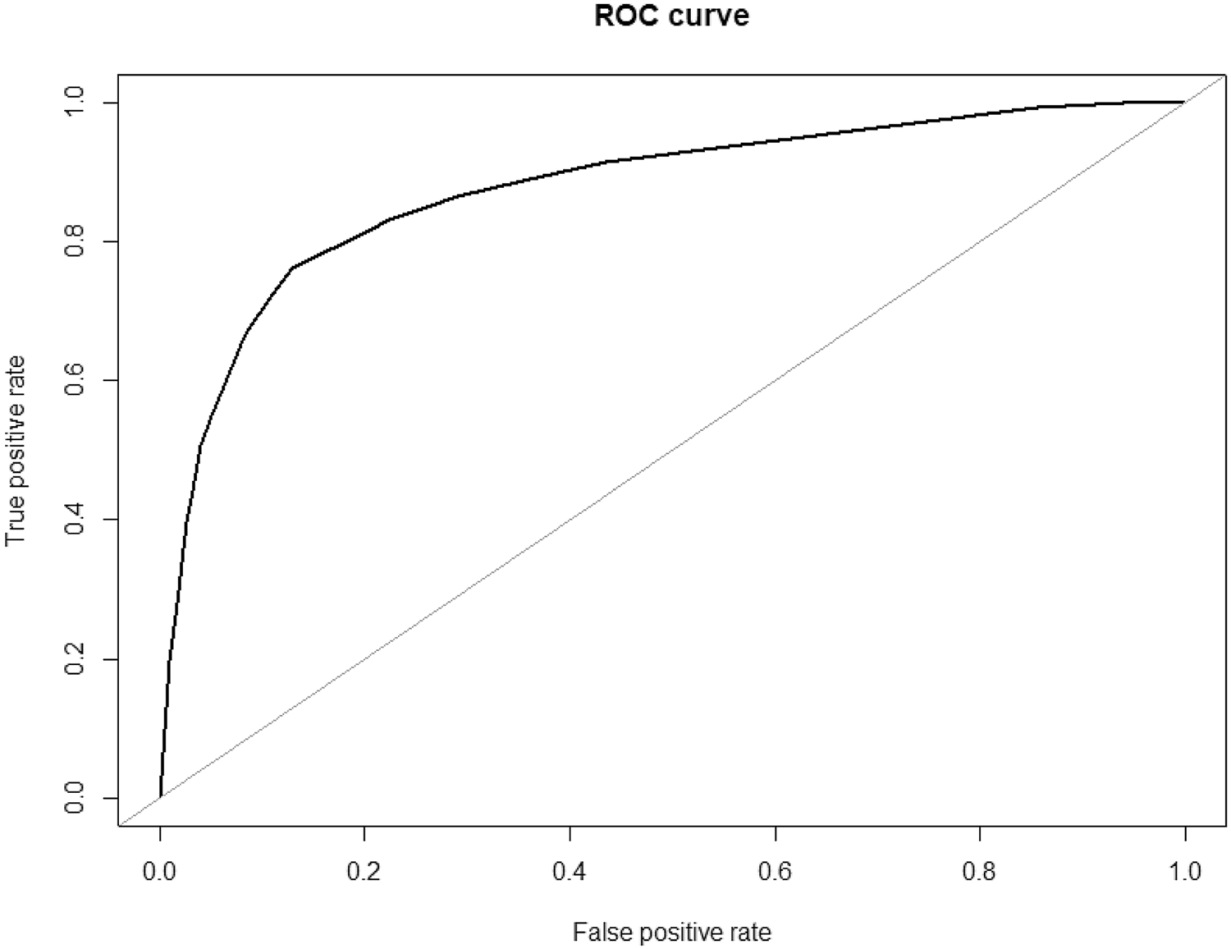
ROC (receiver operating characteristics) curve of data (train:test = 80:20) prediction after ROSE oversampling method. AUC was 0.89. R program version 3.6.3 (The R Foundation for Statistical Computing, Vienna, Austria) was used in creating diagram.

**Figure 2:**
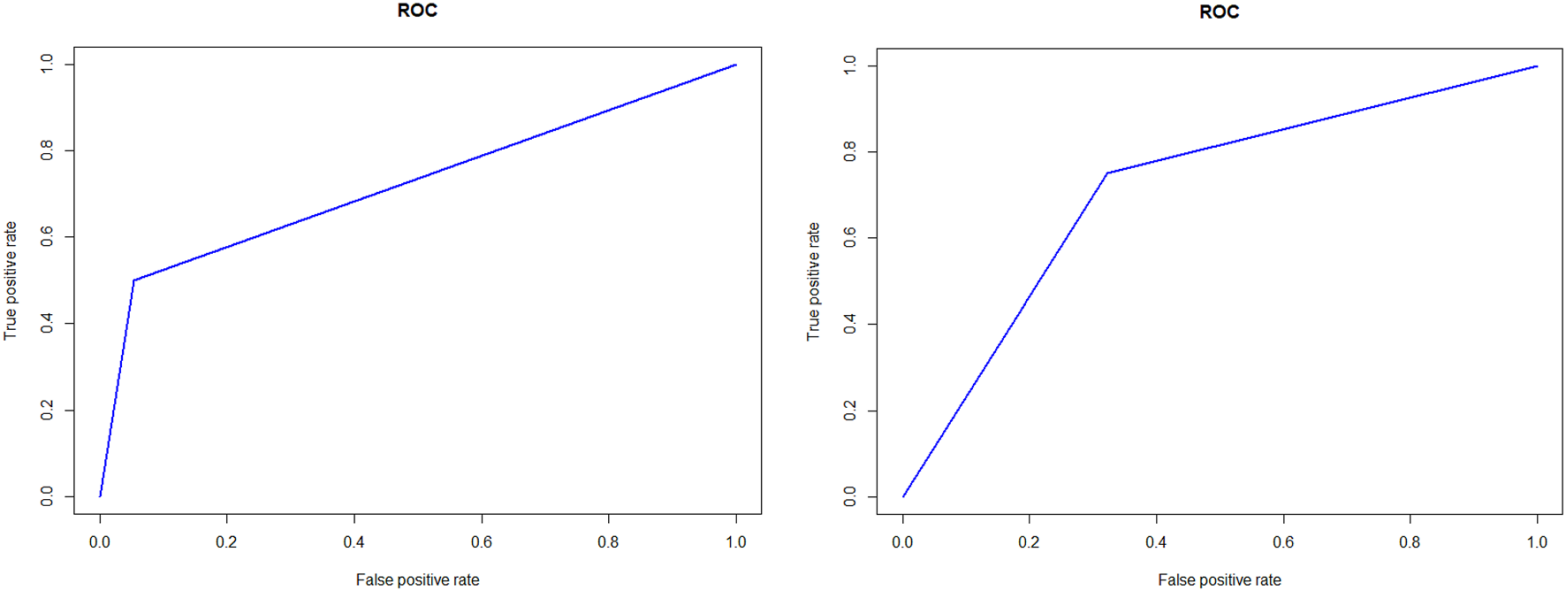
(Left) ROC (receiver operating characteristics) curve of neuralnet prediction package. AUC was 0.79. (Right) ROC (receiver operating characteristics) curve of nnet prediction package. AUC was 0.75. R program version 3.6.3 (The R Foundation for Statistical Computing, Vienna, Austria) was used in creating diagram.

**Figure 3:**
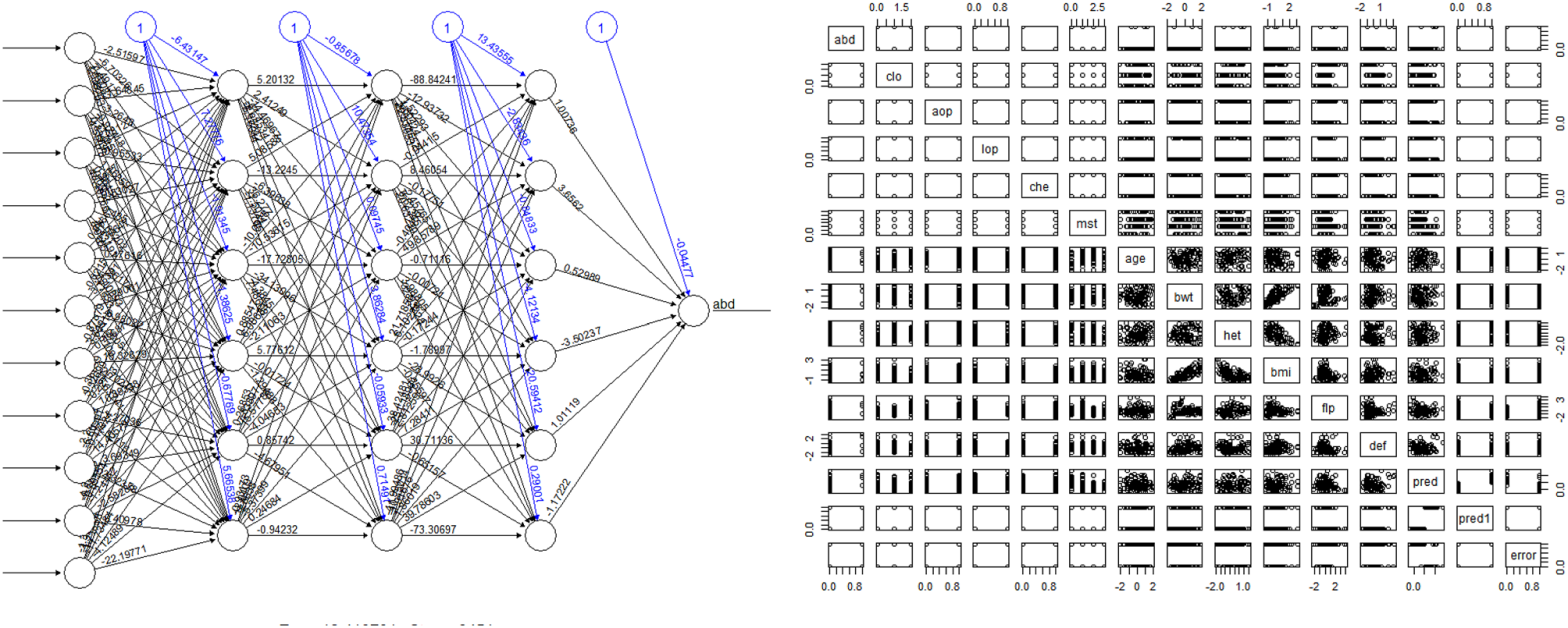
Plot diagram of highest predictive neuralnet function (left) and the result (right). There were six hidden neurons each involved in three hidden layers, resilient backpropagation with backtracking algorithm, sum of squared errors for error calculation, and logistic function for smoothing the result. Maximum steps were set at 10^6^. R program version 3.6.3 (The R Foundation for Statistical Computing, Vienna, Austria) was used in creating diagram.

After ROSE oversampling, the AUC obtained through the test run was 0.89 (Fig. 1). Training and testing sets were divided in an 80:20 ratio, with each training set pre-processed through sMOTE oversampling. The prediction score, sensitivity, specificity, and AUC as measured by the neuralnet, nnet, and RSNNS packages were summarized in Table 2. Among the three packages, the prediction accuracy of the neuralnet package was the best at 81%, and the model accuracy was also the highest (Fig. 2). The prediction rate was the best in the neuralnet prediction function, when the hidden layers were set to 3 layers (Fig. 3) and the reverse propaganda algorithm was used.

**Table 2:** Prediction scores and AUC of each neural network packages.

Package		Proportion(n)	AUC
ROSE	Train	80 (453)	0.89
	Test	20 (114)	
	Train	60 (340)	0.67
	Test	40 (227)	
		Percentage/value	
Neuralnet^a^	Accuracy	81%	0.82
	Sensitivity	0.79	
	Specificity	0.89	
nnet^a^	Accuracy	74%	0.76
	Sensitivity	0.75	
	Specificity	0.92	
RSNNS^a^	Accuracy	69%	0.71
	Sensitivity	0.67	
	Specificity	0.76	

^a^Data was divided into train : test = 80:20.

**Figure 4:**
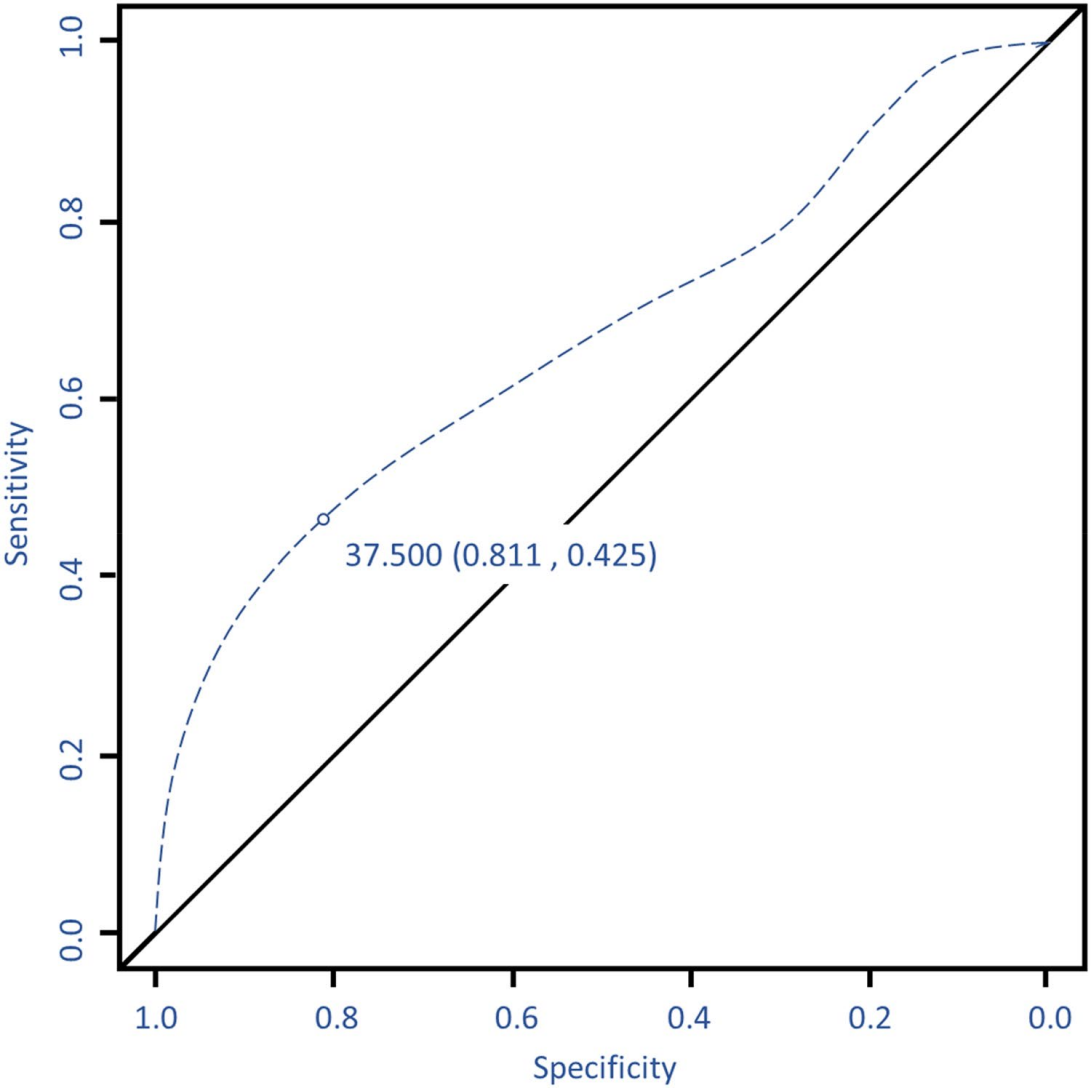
Abdominal fascial defect cutoff value for risk of donor related complication. 37.5 cm^2^ value contained estimated sensitivity of 0.81 and specificity of 0.43. R program version 3.6.3 (The R Foundation for Statistical Computing, Vienna, Austria) was used in creating diagram.

**Figure 5:**
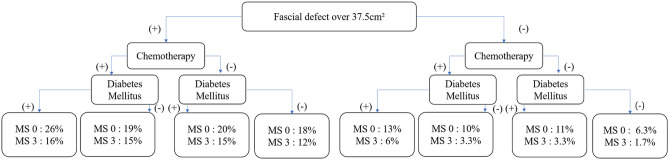
Different probabilities of abdominal complications after autologous breast reconstruction with muscle sparing TRAM flap. The maximal risk with every risk factor present was calculated as 26%, and minimal risk in none of risk factors present was 1.7%.

The cutoff point for maximal sensitivity and specificity regarding complication risk for fascial defect size was 37.5 cm^2^ (Fig. 4). The predicted probability of abdominal complication between the high- and low-risk group was calculated using the highest prediction function of neuralnet package. The high-risk group was designated as: having a fascial defect size of 37.5 cm^2^ or more, having chemotherapy, have history of diabetes mellitus, and performing muscle sparing type 0 procedure. The probability of abdominal complication risk for these patients was 26%. In contrast the low-risk group had: a fascial defect size less than 37.5 cm^2^, no adjuvant chemotherapy, without diabetes, and muscle sparing type 3 surgery, with a probability of 1.7% for abdominal complications. Additionally, different probabilities of abdominal complications in each situation based on risk factors were categorized (Fig. 5).

## Discussion

This study is the first, to our knowledge, to implement a prediction model utilizing machine learning methods for the risk assessment of complications in breast reconstruction with abdominal free flap. The authors applied an efficient prediction model using a neural network technique. Though complications with abdominal free flaps are rare and studies on them are relatively easy to neglect, once it occurs, these donor-related complications give patients and surgeons significant clinical burden^23–25^. In this study, we aimed to present accurate information for more effective risk assessment by patients and doctors.

Breast reconstruction with abdominal flap has evolved from initial methods of harvesting whole muscle, to muscle sparing and DIEA perforator-based flap. Efforts have been made to minimize the amount of tissue harvested from the donor site to reduce postoperative functional deficit and to reduce donor site related complications. Donor-related morbidity is known to be related to the amount of tissue that is harvested from the abdomen during flap elevation^5^. However, even in muscle sparing or DIEP flap elevation procedures, too many perforators can be harvested, leading to abdominal fascia and rectus muscle disruptions, especially in cases when a larger flap volume is needed or when bilateral breast reconstruction is performed. Harvesting perforators from both medial and lateral rows to reduce fat necrosis was shown to lead to more fascial defect^10^. Techniques using single perforator or single row perforator have their own drawbacks as it was shown that the frequency of fat necrosis was high with long-term flap volume decrease after these surgeries^10,26,27^.

This study revealed that prediction accuracy can be efficiently maintained (AUC 0.89) by sMOTE oversampling using the ROSE package of the R program, overcoming severe data imbalances due to low complication rates. Also, although the patient cohort size that was used in this study was not comparably large, the integrity of data after oversampling was high enough to establish neural network models. Among the three neural network packages utilized, neuralnet package outperformed the other two in every category including prediction scores, AUC of ROC curve, sensitivity, and specificity. An impressive prediction accuracy could be obtained (81%) through neuralnet function when setting three different hidden layers and binary classification output. Lastly, use of parallelized capabilities of multi-layer perception was assumed to fail to overperform compared to other two packages. The RSNNS package was the weakest in performance and adding more hidden layers in the settings did not result in better prediction scores (Fig. 5).

The lesson to be learned from this study is that important factors that significantly increase risk for donor-related complications in the muscle sparing tram flap were: defect size at flap elevation, adjuvant chemotherapy, history of diabetes mellitus, and muscle sparing technique type. Notably, the statistical cutoff point for defect size that significantly increases the complication risk is 37.5 cm^2^. Of course, it is better to minimize defects in all surgeries, but when performing surgery in the real-world practice, the position of the lateral and medial row perforators is different for each patient and for each race. Further, the width of the rectus muscle may differ, and the defect size is dependent on the volume of the flap being elevated. This data provides information that can be used as an effective reference for accurately discriminating the risk before and after surgery up to the follow-up period for each patient.

**Figure 6:**
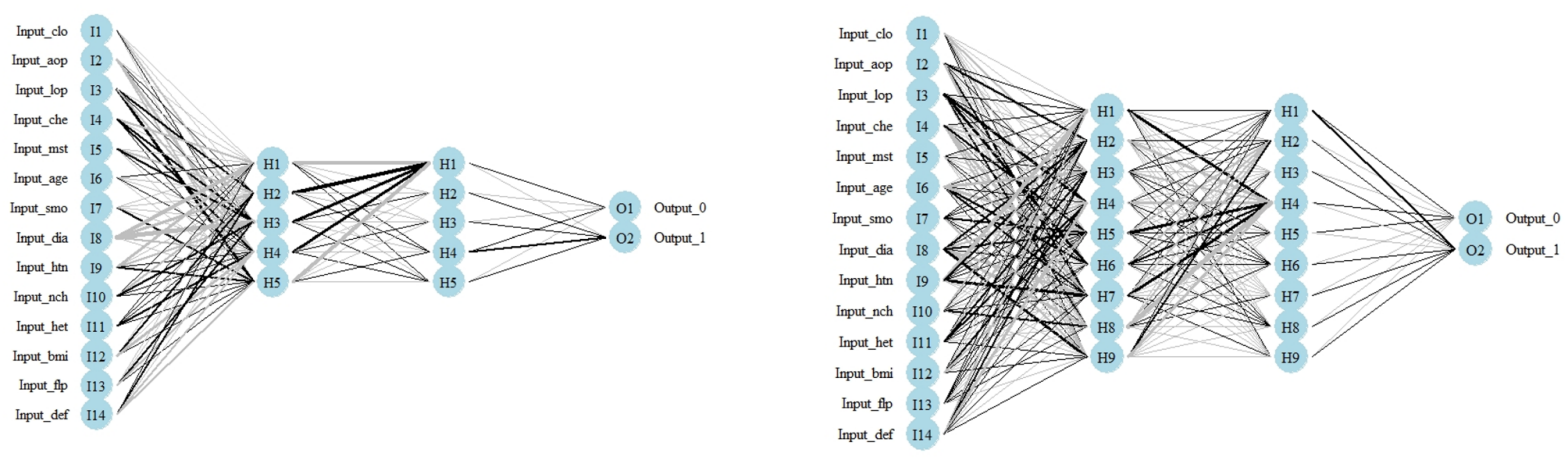
Plot diagram of RSNNS function, with five hidden neurons (left) and nine hidden neurons (right). Different options were tested with RSNNS package, however failed to give satisfying prediction values. R program version 3.6.3 (The R Foundation for Statistical Computing, Vienna, Austria) was used in creating diagram.

RSNNS (R Using Stuttgart Neural Network Simulator) performed lower than classical machine learning packages, neuralnet and NNET. The main strength of RSNNS package is that it has various neural network and visualization function with unlimited ability to increase perceptron layers in utilizing variable datasets (Fig. 6). However, in multi-layered perceptron, hidden layer between input and output layer can not define the standard value of hidden layer output, without backpropagation that was utilized in both neuralnet and NNET packages. The feedforward neural network is assumed to underperform under prefabricated data with ROSE oversampling. However, different training sets with various implementation details should be utilized in order to maintain the utmost method in processing oversampled data, such as abdominal complication after autologous breast reconstruction.

In addition, this study was the first in this field to create a most accurate deep learning prediction model, which was analyzed and validated by using imbalanced data, corrected through sMOTE oversampling, and utilizing three representative packages among neural network machine learning algorithms. Due to the nature of machine learning prediction models, the results may vary depending on the quality and quantity of the data, but by defining the method and package settings used to obtain the most efficient and high AUC when using a prediction model with limited and imbalanced data like in the reconstructive surgical field, these models can be used and replicated effectively for risk assessment of patient outcomes in free flap operation as well as other reconstructive surgical procedures.

The authors note that this study had several limitations encountered. First, it was difficult to specifically define abdominal donor-related complication as there are no exact diagnostic criteria or standards, and among patients with symptoms, not all patients underwent further imaging workup or had hernia repair operation during their variable follow up time span. The most important diagnostic finding to confirm presence of complication was bulging of the surgical site when the patient was in the upright position. Since the patient's symptomatic complaint and the physical examination of the surgeon were the most accurate diagnostic measure, we used these to determine occurrence of donor-related complications for this retrospective review. However, in all cases where the patient complained of having abdominal hernia or bulging, the responsible surgeon always performed physical examination to confirm it, and majority of the cases were radiographically confirmed with abdominal CT or sonography. In addition, the cases that were primarily diagnosed by the surgeon during follow-up period without primary complaints from the patients were also included.

In addition, the possibility that there are additional risk factors, other than the 15 factors investigated in this study, cannot be excluded. According to previous studies, factors other than those analyzed in this study may be responsible for abdominal donor-related complication inducing: harvested perforator vessel number and denervation of intercostal nerve^4–7,11,12^. There were, likewise, several other factors that could have been considered, such as: how long the surgery took, whether there was a flap-related complication after surgery, the extent of blood loss during surgery, and the duration of hospitalization. To facilitate efficient operation of the risk prediction model through supervised learning however, we chose a method of organizing the data by narrowing it down to several more likely factors. In addition, in the process of simplifying data, clustering and normalization of data was performed and data mining was unified for various factors as a result, such as: more specific types/sizes of mesh used for fascia closure and data on the manufacturer or the size of acellular dermal matrix that was used for fascia closure. Although this data was clustered, it is impossible to completely rule out the possibility that each subfactor could be independently responsible for various consequences in the patient's recovery process after surgery. For these limitations, it is necessary to analyze individual factors more effectively through further studies on risk assessment.

We admit that there are inherent limitations to this type of research, and that various efforts have been involved to find effective triggers through many previous studies. Many studies on how to select key variables that affect final prediction outcome in order to execute machine learning studies have been conducted using existing classical statistical methods, not ML packages, or by simply referring to previous references^17,28,29^. However, our study acknowledged such limitations and focused on providing more efficient information by applying the advanced data processing model to patients and physicians in the treatment process before and after the surgery. Logistic multivariate regression in fact is also a kind of statistical prediction model. It was used to screen and select which factor is responsible in affecting the final outcome. If every clinical variable could have been objectively investigated and analyzed accurately and completely, it would have been a perfect thesis. However, the technical difficulties regarding data processing remains, and the authors, to our knowledge, chose the most efficient way in processing the clinical data regarding such a complicated, multi-factorial, overfitted and imbalanced breast reconstruction outcome data.

## Conclusion

To create a more accurate and efficient deep learning-based prediction model of donor-related complications in autologous breast reconstruction, we overcame various limitations inherent to unequal data, and validated the predictive accuracy between three representative neural network packages. This method of risk assessment for rare, but troublesome complications through a machine learning approach, performed by the authors for the first time, can potentially be replicated and used for studying complications in other procedures. Using this model, defect size at flap elevation, adjuvant chemotherapy, and muscle sparing technique type were found to be factors that significantly increase risks for post-operative complications. These results may help surgeons to achieve better surgical outcomes and reduce postoperative burden (Supplementary information S1 and S2).

## Supplementary Information

Supplementary Information 1.

Supplementary Information 2.
